# The Effectiveness and Safety of Fluoroquinolone-Containing Regimen as a First-Line Treatment for Drug-Sensitive Pulmonary Tuberculosis: A Systematic Review and Meta-Analysis

**DOI:** 10.1371/journal.pone.0159827

**Published:** 2016-07-25

**Authors:** Hyun Woo Lee, Jung Kyu Lee, Eunyoung Kim, Jae-Joon Yim, Chang-Hoon Lee

**Affiliations:** 1 Division of Pulmonary and Critical Care Medicine, Department of Internal Medicine, Seoul National University College of Medicine, Seoul National University Hospital, Seoul, 03080, Republic of Korea; 2 Division of Pulmonary and Critical Care Medicine, Department of Internal Medicine, Seoul Metropolitan Government-Seoul National University Boramae Medical Center, Seoul, 07061, Republic of Korea; 3 Department of Statistics, Kyungpook National University, Daegu, 41566, Republic of Korea; Azienda ospedaliero-universitaria di Perugia, ITALY

## Abstract

**Background:**

Fluoroquinolone is recommended as a pivotal antituberculous agent for treating multi-drug-resistant pulmonary tuberculosis. However, its effectiveness as first-line treatment remains controversial. The present study was conducted to validate the fluoroquinolone-containing regimen for drug-sensitive pulmonary tuberculosis.

**Methods:**

We searched MEDLINE, EMBASE, and the Cochrane Central Register of Controlled Trials until June 5, 2015. Randomized controlled trials (RCTs) that compared antituberculous regimens containing fluoroquinolone with the standard regimen were included.

**Results:**

Eleven RCTs that included 6,334 patients were selected. Fluoroquinolone-containing regimens had a higher rate of sputum culture conversion at 2 months of treatment (M-H fixed odds ratio [OR], 1.36; 95% confidence interval [CI], 1.20–1.54). However, the outcomes were less favorable (M-H fixed OR, 0.69; 95% CI, 0.59–0.82) and the associated total adverse events were more frequent (M-H fixed OR, 1.84; 95% CI, 1.46–2.31) in the fluoroquinolone-containing regimen group, without a significant heterogeneity according to treatment duration. Treatment with the fluoroquinolone-containing regimen for 4 months showed a higher relapse rate.

**Conclusions:**

Despite a higher culture conversion rate at 2 months of treatment, the fluoroquinolone-containing regimen had limitations, including less favorable outcomes and more adverse events, as the first-line therapy for drug-sensitive pulmonary tuberculosis.

## Introduction

Pulmonary tuberculosis (TB) is a contagious disease in which the human lung is primarily infected by a pathogen, *Mycobacterium tuberculosis*. According to the World Health Organization (WHO) report from 2014, 9 million occurrences and 1.5 million deaths of TB were found worldwide in 2013 [1]. Since the Stop TB Partnership was established in 1998, the prevalence of TB and mortality from TB slowly declined. It is estimated that about 37 million patients were saved through appropriate treatment such as the standard regimen of isoniazid (H), rifampin (R), ethambutol (E), and pyrazinamide (Z) (HREZ) with a successful treatment rate as high as from 86–95% [1,2].

Adherence to the treatment for drug-sensitive pulmonary TB is one of the most important factors for maximizing the efficacy of TB treatment and minimizing the occurrence of multiple drug resistance [3]. Patients’ poor adherence to anti-TB therapy contributes to treatment failure, relapse, or death by pulmonary TB [4,5]. Therefore, numerous efforts have been made to improve the tolerance of patients to anti-TB medication by decreasing the treatment duration [6,7]. However, we still use HREZ for 6 months as a treatment of choice.

Fluoroquinolone is one of the anti-TB agents with a highly effective early bactericidal activity [8,9]. Studies have reported on the effectiveness and importance of fluoroquinolone for the successful treatment of multi-drug resistant pulmonary TB [10,11]. Fluoroquinolone also has been reported to have significant effectiveness on drug-sensitive pulmonary TB as first-line treatment. Previous randomized controlled trials (RCTs) have shown that ofloxacin-containing regimens can replace the HREZ regimen and reduce treatment duration [12,13]. Other fluoroquinolones such as gatifloxacin and moxifloxacin had good treatment effectiveness with an excellent sputum mycobacterial culture conversion rate at 2 months [14,15].

However, no significant differences in the sputum culture conversion rate at 2 months were found between the fluoroquinolone-containing regimen and standard regimen in a previous systematic review and meta-analysis [16,17]. Those studies did not include recent RCTs in which the fluoroquinolone-containing regimens with gatifloxacin or moxifloxacin and treatment duration of 4 months [18–20]. In addition, a meta-analysis to evaluate adverse events of various fluoroquinolones has not been sufficiently conducted.

Thus, we conducted a systematic review and meta-analysis that included recent trials [18–22] to evaluate the effectiveness and safety of the fluoroquinolone-containing regimen in the treatment of drug-sensitive pulmonary TB.

## Materials and Methods

For the present systematic review and meta-analysis, we followed the Preferred Reporting Items for Systematic Reviews and Meta-Analyses guidelines [23].

### Search Strategy and Selection Criteria

We searched the MEDLINE, EMBASE, and Cochrane Library databases (search date: June 5, 2015). The search terms were “tuberculosis,” “quinolone,” and “randomized protocol design.” Quinolones included ofloxacin, levofloxacin, moxifloxacin, and gatifloxacin. Details of the search strategy are shown in the S1 File.

The inclusion criteria were as follows: 1) RCTs; 2) studies that included patients older than 18 years with newly diagnosed pulmonary TB that was microbiologically confirmed without evidence of resistance to rifampicin or fluoroquinolone; 3) studies that compared fluoroquinolone-containing regimens with standard therapy; and 4) published clinical trials and abstracts in the English language. Standard therapy was defined as HREZ for a 2-month intensive treatment period and HR for a 4-month maintenance treatment period or HRE for a 2-month intensive treatment period and a 7-month maintenance treatment period.

### Data Extraction and Assessment of the Risk of Bias

Two authors independently checked and selected studies based on titles and abstracts, in accordance to the inclusion criteria. They were blinded to the results of each report and excluded studies that did not meet the inclusion criteria, were duplicated, or were inaccessible. Any disagreement regarding the inclusion or exclusion of studies was resolved by referring to the original articles and discussing them as a group.

The risk of bias of each study was assessed in eight dimensions by applying the Cochrane risk of bias tool (S2 File) as follows: 1) selection bias by adequacy of random sequence generation and allocation concealment; 2) performance bias by appropriate blinding of participants and researchers; 3) attrition bias by knowing if missing data were absent, the reason of exclusion after randomization was not relevant to the study result, and the number and reason of missing data were similar between regimens; 4) reporting bias by reviewing the study protocol and checking the funnel plot; and 5) other biases. As the risk of bias was varied between the efficacy outcomes and safety outcomes in the same study, we applied different weights to each outcome regarding performance and attrition biases. The researchers discussed any disagreement together and reached a consensus.

The collected baseline data from the extracted studies were as follows: author, published year, number of study subjects, inclusion and exclusion criteria, and characteristics of the enrolled population. We determine the characteristics of the enrolled patients, such as sex, smoking status, country, ethnicity, human immunodeficiency virus (HIV) infection status, and existence of pulmonary cavity. We also determined the kinds of medicines used and the treatment duration in each study.

The primary outcome was the sputum culture conversion rate at 2 months of treatment between the fluoroquinolone-containing and standard regimens. Secondary outcomes were treatment failure, relapse, total favorable outcomes, adverse events, serious adverse events, mortality, and adherence. Sputum culture conversion at 2 months of treatment was defined as a negative sputum mycobacterial culture at the end of intensive treatment during the initial 2 months. Treatment failure was defined as at least two consistent positive sputum culture results after 2 months of anti-TB treatment. We determined relapse as a positive conversion of sputum mycobacterial culture after a favorable response at the end of treatment without evidence of reinfection. Total favorable outcome was defined as a negative sputum mycobacterial culture at the end of the whole anti-TB treatment without any outcomes classified as unfavorable. Unfavorable outcomes were events including treatment failure, relapse, death, and any schedule change in treatment due to medical problems, except pregnancy or the initiation of antiretroviral treatment. An adverse event was defined as any newly developed side effect after receiving anti-TB medication. The adverse events were analyzed separately. Gastrointestinal adverse events consisted of nausea, vomiting, abdominal pain, and diarrhea. Drug reactions included drug fever and skin rash. Serious adverse events were considered life-threatening conditions (grade IV), an anti-TB treatment regimen change, hospitalization, or death. Peri-treatment mortality included all-cause deaths during treatment and follow-up. Adherence was defined as compliance to >80% of the planned treatment of the prescribed regimen.

### Statistical analysis

Meta-analysis was used to estimate the odds ratio (OR) with the 95% confidence interval (CI) for the outcome data by using the Mantel-Haenszel (M-H) methods and the fixed effect model. The Q-statistic method with the chi-square test was performed to identify and measure heterogeneity among the pooled data. Heterogeneity among the studies was assumed when the p value was <0.1. The *I*^2^ statistics was calculated for quantification of inconsistency among studies to compensate for the limitation of the chi-square test. If the *I*^2^ was >30%, we considered verifying the meta-analysis result by using the random-effects model. We described the *I*^2^ value first and then the p value by using the chi-square test. We conducted subgroup analyses on the following: 1) trials of fluoroquinolone-containing regimens for 6 months compared with 4 months, 2) trials of adding fluoroquinolone to the standard regimen compared with trials that replaced ethambutol with fluoroquinolone and trials that replaced isoniazid with fluoroquinolone, and 3) trials with HREZ as the standard regimen. A sensitivity analysis was performed to assess the effect of a later-generation fluoroquinolone in each meta-analysis (i.e., moxifloxacin, gatifloxacin, and levofloxacin). Publication bias was assessed by using a funnel plot. All the analyses were performed with Review Manager version 5.3 (Copenhagen: The Nordic Cochrane Center, The Cochrane Collaboration, 2014).

## Results

**Fig 1:**
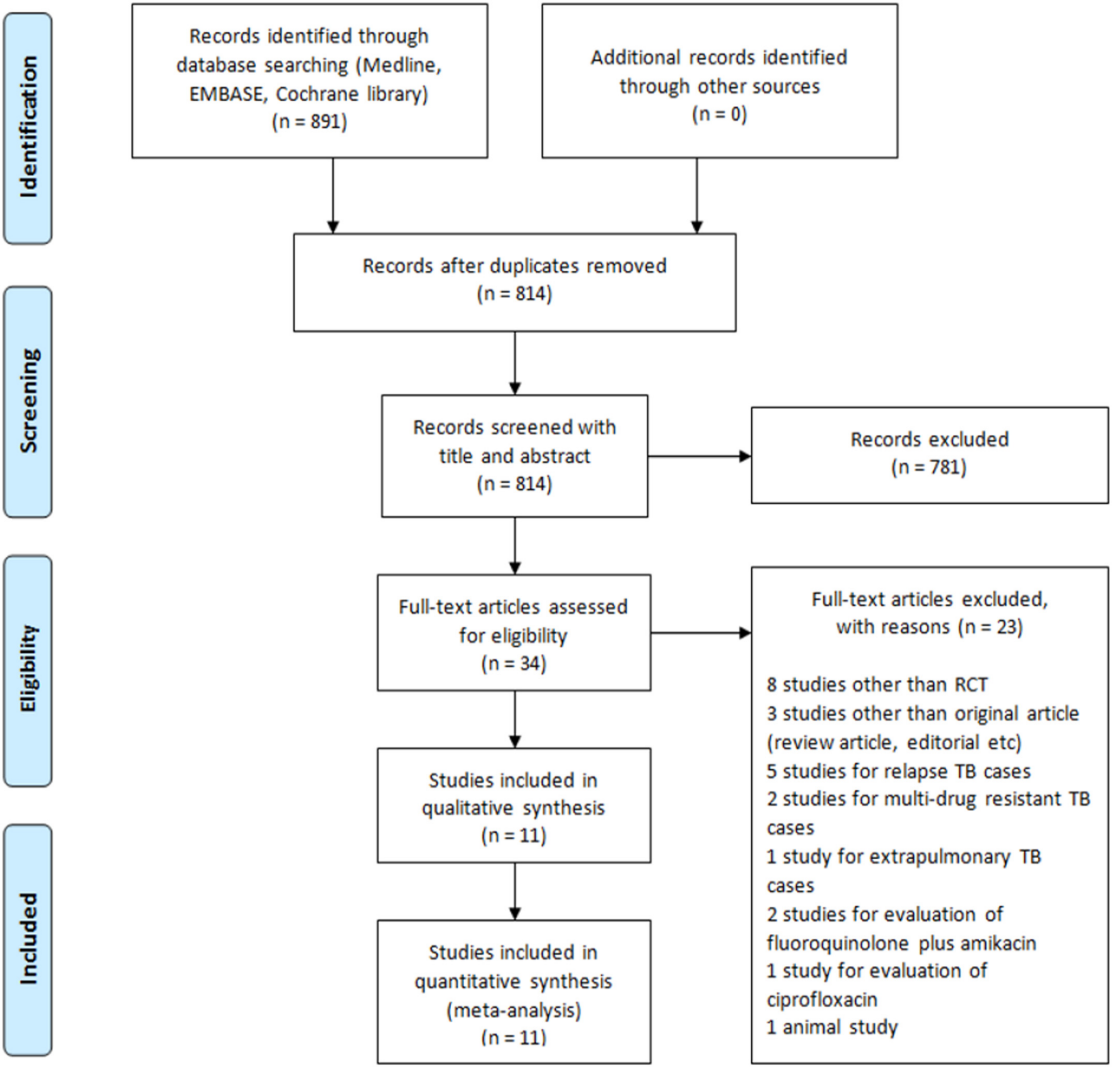
PRISMA flow chart for the meta-analysis.

We found 739 studies in MEDLINE, 42 in EMBASE, and 110 in the Cochrane Library by searching for the key terms in titles and abstracts. We found 77 duplicated studies. We excluded 781 studies that were not suitable according to the study objective. After reviewing the full text of 34 studies, we finally selected 11 that met the inclusion criteria (Fig 1). After data extraction, the risk of bias was assessed according to 10 areas of bias items (S1 Fig) with PRISMA checklist (S3 File).

### Baseline characteristics of patients in the included studies

The 11 RCTs included 6,334 patients enrolled from 1992 to 2014. We found the following three types of interventions: fluoroquinolone added to standard therapy in 2 studies [22,24], fluoroquinolone replaced ethambutol in 7 [12,14,15,19–21,25], and fluoroquinolone replaced isoniazid in 3 [18,20,26]. Each study had anti-TB treatment durations of 4, 6, or 9 months. We found several types of fluoroquinolones used as an intervention as follows: moxifloxacin in 8 studies, gatifloxacin in 3, ofloxacin in 2, and levofloxacin in 1 (Table 1). Control therapy was used as the standard HREZ regimen for 6 months, except in 1 study that used an HRE regimen for 9 months. All the control standard regimens were administered for a longer duration. Most of the patients were men, ranging from 62% to 77% of the study subjects, and non-white patients. Eight studies included HIV and non-HIV patients, 1 included only HIV patients, 1 excluded HIV patients, and 1 did not evaluate the patients’ HIV status. HIV-seropositive status was confirmed in 1,024 patients, and pulmonary cavitary lesions were found in 3,655 patients.

**Table 1:** Baseline characteristics of each included study.

Study	Published year	Type of fluoroquinolone used in the intervention	Standard regimen	Male sex	Smoking status	Region	HIV status	Cavity
Kohno et al.[12]	1992	Ofloxacin	HRE	69%	No remark	Japan	No remark	80%
El-Sadr et al.[24]	1998	Levofloxacin	HREZ	77%	No remark	United States	100%	10%
Burman et al.[25]	2006	Moxifloxacin	HREZ	67%	No remark	Africa, North America	22%	74%
Rustomjee et al.[14]	2008	Gatifloxacin, Moxifloxacin, Ofloxacin	HREZ	67%	No remark	Africa	59%	94%
Dorman et al.[26]	2009	Moxifloxacin	HREZ	72%	Never smoked: 60%	North America, Brazil, Africa, Spain	11%	76%
Conde et al.[15]	2009	Moxifloxacin	HREZ	62%	Never smoked: 55%	Brazil	3%	68%
Jawahar et al.[21]	2013	Moxifloxacin, Gatifloxacin	HREZ	74%	No remark	India	No remark	No remark
Velayutham et al.[22]	2014	Moxifloxacin	HREZ	75%	No remark	India	All excluded	36%
Merle et al.[19]	2014	Gatifloxacin	HREZ	73%	No remark	Africa	18%	61%
Jindani et al.[18]	2014	Moxifloxacin	HREZ	64%	Never smoked: 49%	Africa	27%	65%
Gillespie et al.[20]	2014	Moxifloxacin	HREZ	70%	Never smoked: 46%	Africa, India, China, Mexico	7%	71%

HREZ = isoniazid, rifampin, ethambutol, and pyrazinamide; HRE = isoniazid, rifampin, ethambutol.

### Effectiveness of replacing the standard regimen with the fluoroquinolone-containing regimen

#### Sputum mycobacterial culture conversion at 2 months of treatment

**Fig 2:**
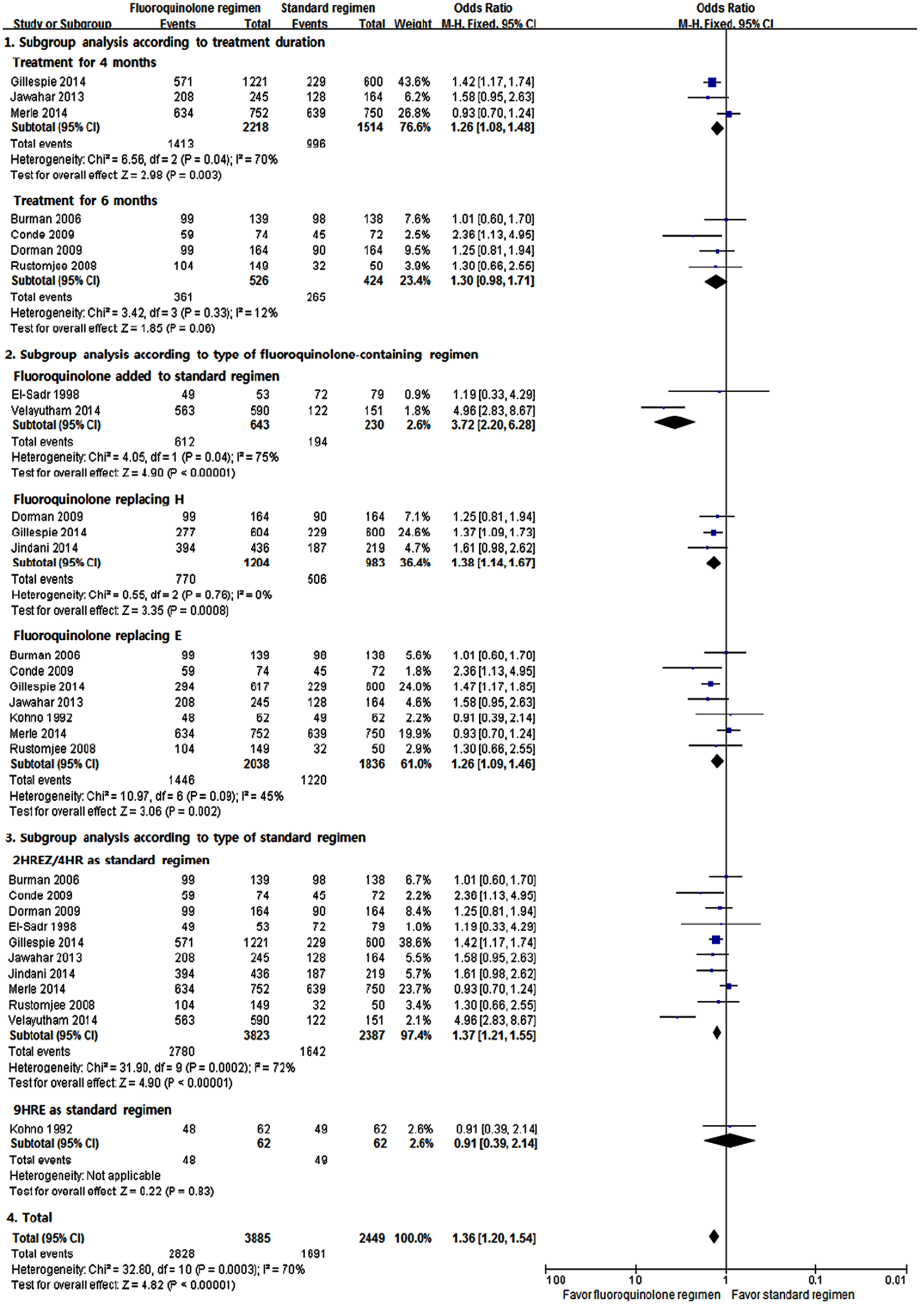
Sputum culture conversion rates at 2 months of treatment. H = isoniazid; R = rifampicin; E = ethambutol; Z = pyrazinamide.

Eleven RCTs were included to compare the sputum culture conversion rate between the fluoroquinolone-containing and standard regimens (Fig 2). The meta-analysis showed the superiority of the fluoroquinolone-containing regimen over the standard regimen in terms of sputum culture conversion rate at 2 months of treatment (M-H fixed odds ratio [OR], 1.36; 95% CI, 1.20–1.54). Although substantial heterogeneity was found (*I*^2^ = 70%, p = 0.0003), at least the superiority of the fluoroquinolone-containing regimen over the standard regimen was consistently observed in the subgroup analyses. The random-effects model yielded similar results (data not shown). We could not find evidence of publication bias in the funnel plot (S2 Fig).

#### Relapse

**Fig 3:**
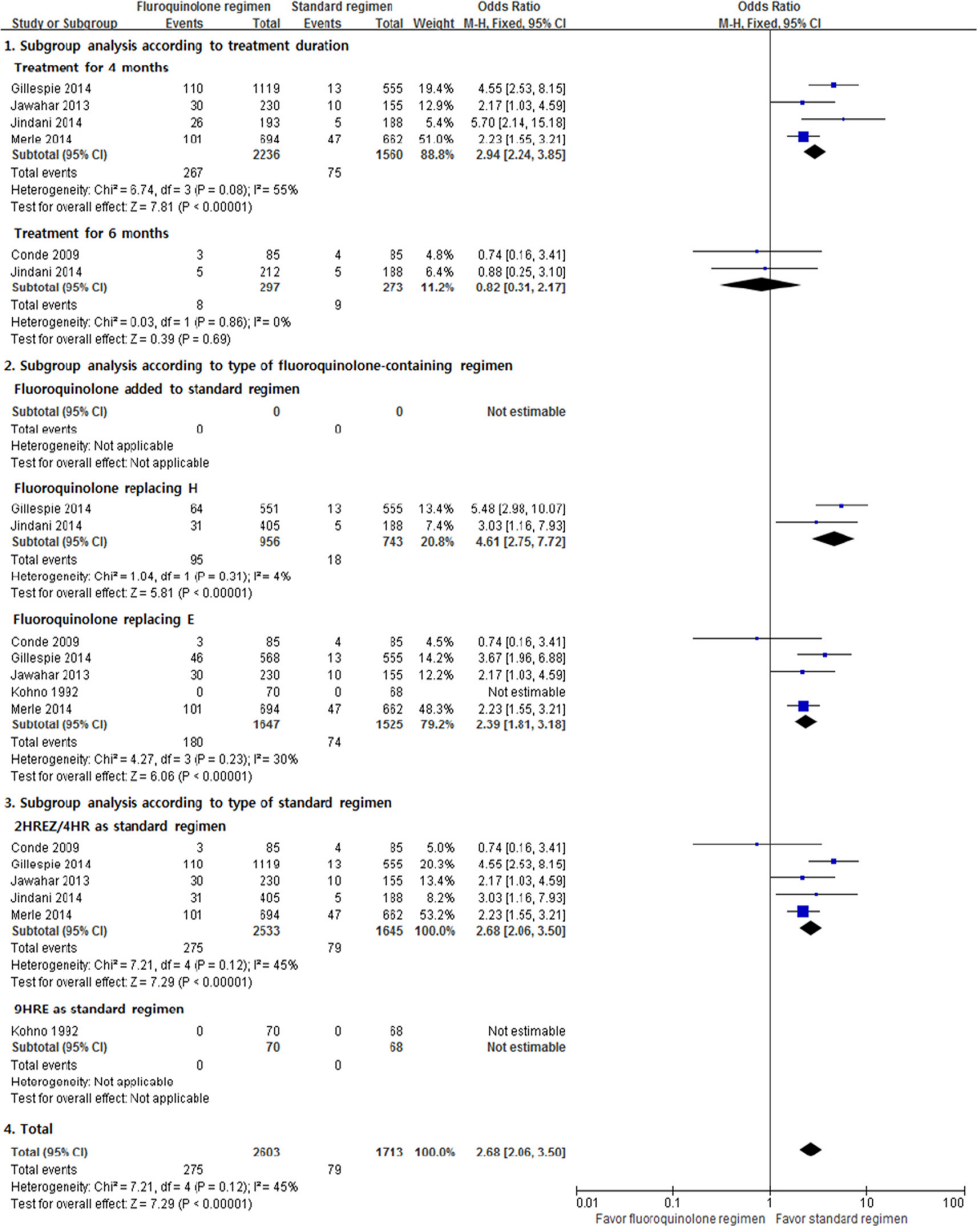
Relapse. H = isoniazid; R = rifampicin; E = ethambutol; Z = pyrazinamide.

Six RCTs were analyzed to determine the differences of relapse between the fluoroquinolone-containing and standard regimens. Significantly more relapses occurred with the fluoroquinolone-containing regimen (M-H fixed OR, 2.68; 95% CI, 2.06–3.50; Fig 3). However, in the subgroup analyses, relapse after 6 months of treatment with the fluoroquinolone-containing regimen did not significantly differ to that with the standard regimen. Although moderate heterogeneity was observed (*I*^2^ = 45%, p = 0.12), the subgroup analyses showed consistent results.

#### Treatment failure

Four RCTs were included in the meta-analysis of treatment failure. The fluoroquinolone-containing regimen did not reduce the treatment failure rate (M-H fixed OR, 0.68; 95% CI, 0.39–1.21; S3 Fig). No heterogeneity was found (*I*^2^ = 0%, p = 0.92), and the results were similar in the subgroup analyses.

### Safety of replacing the standard regimen with the fluoroquinolone-containing regimen

#### Total favorable outcomes

**Fig 4:**
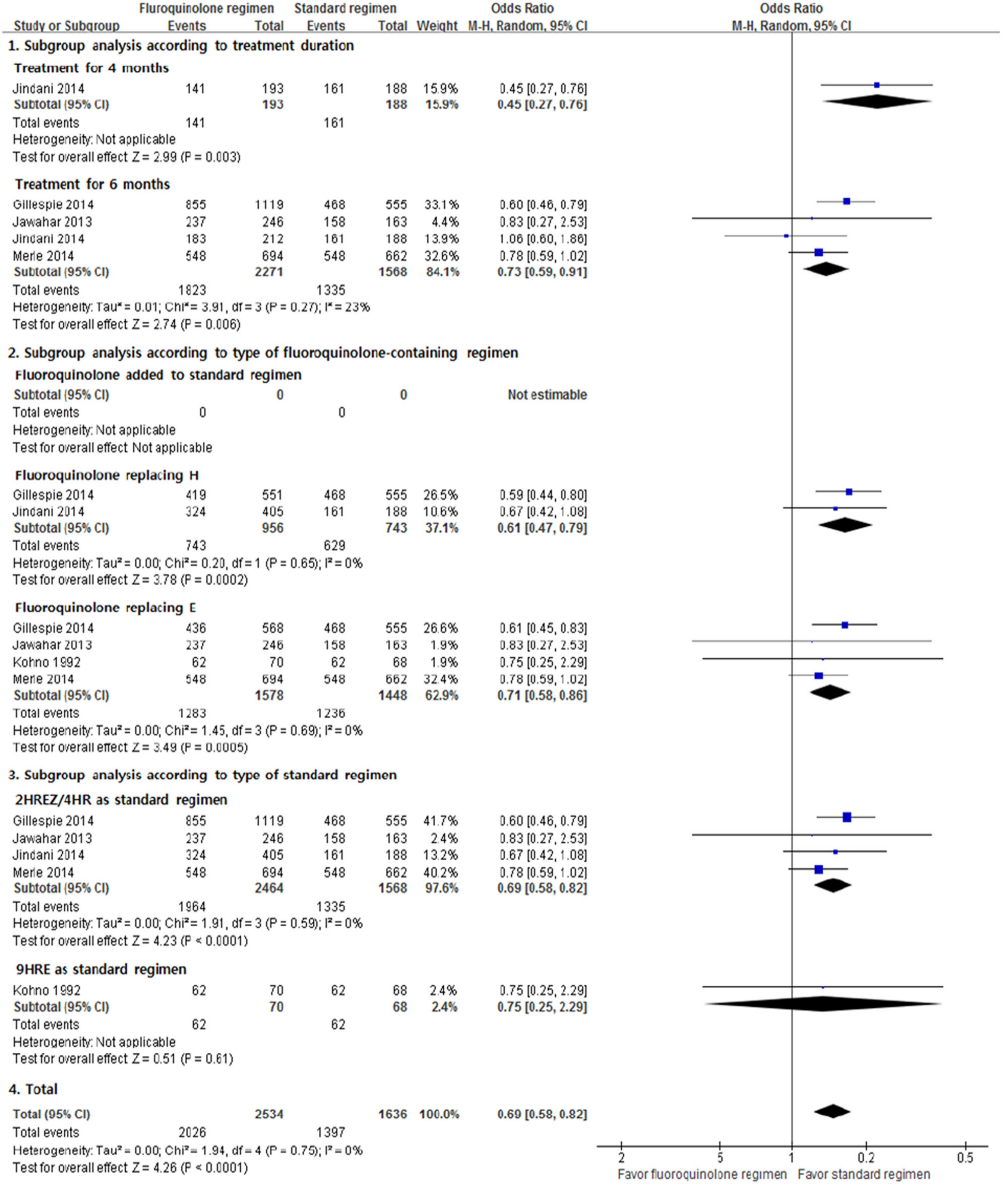
Total favorable outcomes. H = isoniazid; R = rifampicin; E = ethambutol; Z = pyrazinamide.

Six RCTs were available for the meta-analysis of total favorable outcomes. The incidence of total favorable outcomes was significantly lower in the fluoroquinolone-containing regimens (M-H fixed OR, 0.69; 95% CI, 0.58–0.82; Fig 4). No heterogeneity was found (*I*^2^ = 0%, p = 0.75), and the results were similar in the subgroup analyses.

#### Adverse events

**Fig 5:**
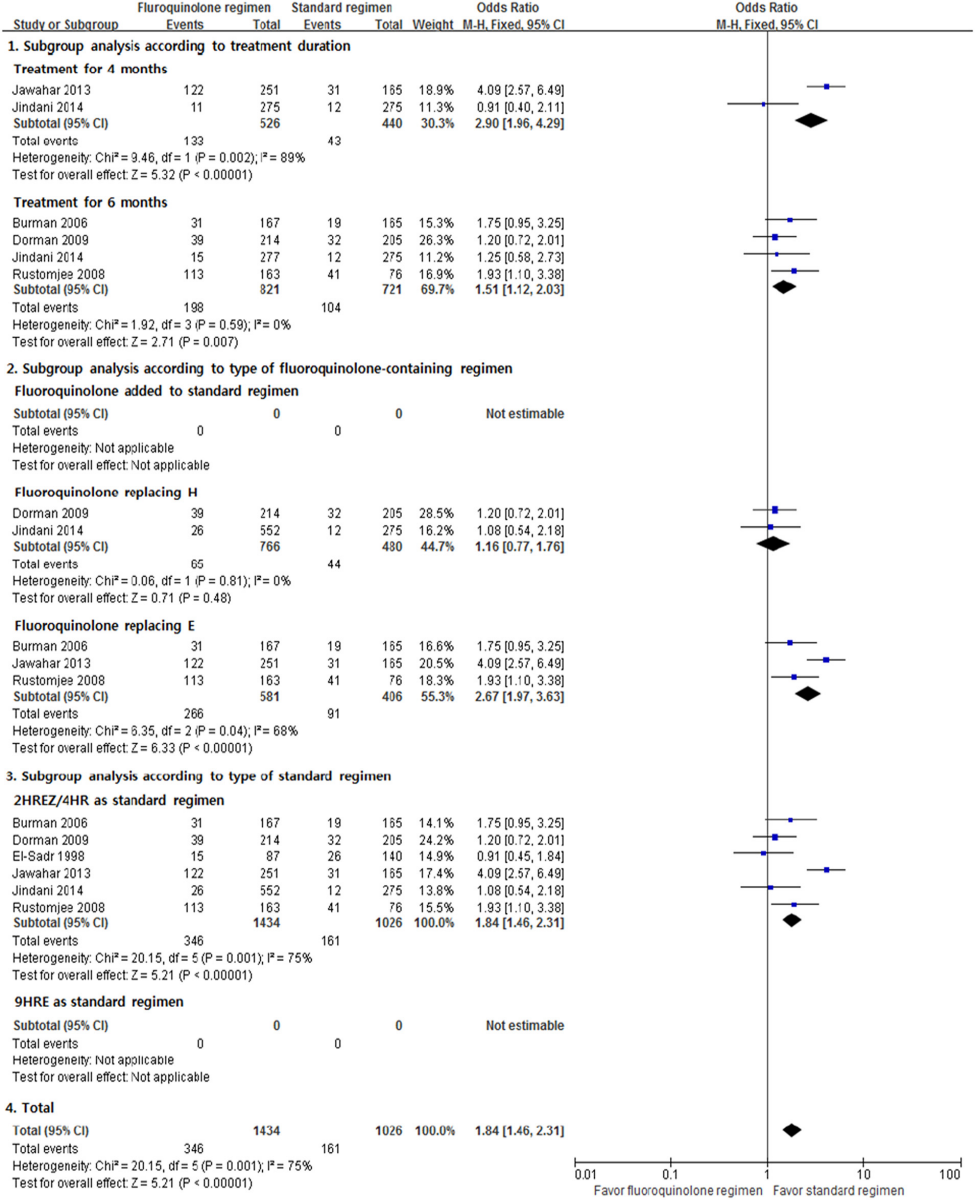
Total adverse events. H = isoniazid; R = rifampicin; E = ethambutol; Z = pyrazinamide.

We used 6 RCTs in the meta-analysis of total adverse events and found considerable heterogeneity among the studies. The result showed that the fluoroquinolone-containing regimen was associated with more adverse events (M-H fixed OR, 1.84; 95% CI, 1.46–2.31; Fig 5). Substantial heterogeneity was found in the evaluation of total adverse events (*I*^2^ = 75%, p = 0.001). In the subgroup analysis, regimens that replaced isoniazid with fluoroquinolone did not cause more adverse events than HREZ.

In the evaluation of hepatotoxicity, drug rash and fever, and serious adverse events, no significant differences were found between the two regimens (S4–S6 Figs). However, gastrointestinal adverse events, dizziness, and joint pain were detected significantly more frequently in the patients that received fluoroquinolone-containing regimens (S7–S9 Figs). Regimens that replaced ethambutol with fluoroquinolone, especially showed a higher OR for the occurrence of several adverse events than other fluoroquinolone-containing regimens.

#### Mortality

No significant difference in peri-treatment mortality was observed between the fluoroquinolone-containing and standard regimens (M-H fixed OR, 0.92; 95% CI, 0.66–1.29; S10 Fig). The observed duration for detecting death was different among the studies. Four studies assessed all deaths that occurred during and after treatment [15,18–20], 1 study evaluated deaths during treatment [26], and 2 studies included only deaths until 2 months of treatment [24,25]. The subgroup analysis showed a similar mortality rate in each group, and statistical heterogeneity was not found (*I*^2^ = 0%, p = 0.84).

#### Adherence

We found no significant difference in treatment adherence between the fluoroquinolone-containing and standard regimens (M-H fixed OR, 1.19; 95% CI, 0.97–1.45; S11 Fig). Heterogeneity was found because 1 study used a stricter definition of adherence than the other studies and reported favorable results of treatment adherence to the gatifloxacin-containing regimen for 4 months (*I*^2^ = 35%, p = 0.09) [19].

#### Effectiveness and safety of later-generation fluoroquinolone-containing regimen

Sensitivity analysis was performed after excluding populations who used ofloxacin and their counterparts in 2 studies [12,14]. A sensitivity analysis revealed that neither the effectiveness nor the safety of a later-generation fluoroquinolone-containing regimen showed obviously different results (data not shown).

## Discussion

The present study showed a significantly higher sputum mycobacterial culture conversion rate at 2 months of treatment with the fluoroquinolone-containing regimen than with the HREZ or HRE regimen. We did not find statistically significant differences in treatment failure and treatment adherence between the fluoroquinolone-containing and standard regimens. However, the fluoroquinolone-containing regimen group showed less favorable outcomes. In addition, more total adverse events were occurred in the fluoroquinolone-containing regimen group. Significantly more cases of gastrointestinal adverse events, dizziness, and joint pain were associated with the fluoroquinolone-containing regimen.

Previous systematic reviews have reported no significant difference in the rate of sputum mycobacterial culture conversion at 2 months of treatment between the fluoroquinolone-containing regimen and standard regimen, whereas relapse or treatment failure was not fully evaluated because of the lack of available studies [16,17]. In terms of safety, only nausea was more frequently associated with the fluoroquinolone-containing regimen [16]. The present study included 5 studies that were evaluated previously and 6 additional studies [12,18–22], and we performed subgroup meta-analyses according to the treatment duration, which has not been done before.

Our study found that fluoroquinolone has a strong anti-TB effectiveness, as evidenced by, for example, a high sputum mycobacterial culture conversion rate at 2 months of treatment, which is consistent with previous results of good early bactericidal effects [8,27]. Sputum mycobacterial culture conversion rate at 2 months of treatment has been regarded as an important surrogate marker of relapse and has been used as a primary outcome [28]. However, considering that more relapses are found in the fluoroquinolone-containing regimen group, a higher sputum mycobacterial culture conversion rate at 2 months of treatment alone cannot guarantee successful TB treatment. A negative sputum mycobacterial culture conversion rate at 2 months of treatment and treatment duration should both be considered predictors of relapse. Previous reports have implied that only a very high sputum mycobacterial culture conversion rate at 2 months of treatment can decrease the relapse rate and treatment duration [28]. According to these studies, a 99% culture-negative conversion rate is necessary to reduce the treatment duration from 6 months to 4 months without relapse of >5%. In our study, the fluoroquinolone-containing regimen showed only a 72.8% sputum mycobacterial culture conversion rate at 2 months of treatment. Fluoroquinolone is not powerful enough to reduce the treatment duration. This finding is also supported by a result that the 6-month fluoroquinolone-containing regimen did not increase the relapse rate. In the present study, the 4-month fluoroquinolone-containing regimen had an 11.9% relapse rate, whereas treatment with fluoroquinolone for 6 months showed a 2.7% relapse rate. The 4- and 6-month fluoroquinolone-containing regimen groups showed less total favorable outcomes regarding treatment failure, relapse, death, and unexpected treatment changes, which suggest that the 6-month fluoroquinolone-containing regimen has limitations in replacing the standard regimen. Although fluoroquinolone-containing regimen with 6-month treatment duration can be considered as an alternative regimen, it is not easy to replace the current standard regimen. Our study showed that the fluoroquinolone-containing regimen is inferior to the standard regimen in terms of adverse events. In addition, previous studies have suggested reserving fluoroquinolone for the future treatment of drug-resistant TB [29,30], and fluoroquinolone is not as accessible and affordable in low-income countries [29].

The strength of this study was in the detection that the fluoroquinolone-containing regimen was superior in terms of the sputum culture conversion at 2 months of treatment and inferior in terms of relapse, which was not demonstrated in previous systematic reviews. In addition, we clarified previous issues regarding adverse events by determining the profiles of each adverse event. Adherence, one of the important factors to achieve successful treatment, was assessed and determined to have no significant difference between the fluoroquinolone-containing regimen and standard regimen.

Several limitations were found in the process of this review. First, significant heterogeneity was found in the meta-analysis for primary outcome. However, all subtotal ORs with a 95% CI were <1·0 in the subgroups, which means that at least fluoroquinolone is superior to the standard regimen according to the 2-month conversion rate. Second, studies that used levofloxacin, which is widely used in the treatment of TB, were few. Third, HRE was included as a standard regimen in this study, which could have led to a lower 2-month conversion rate in the standard regimen group. However, the sensitivity analysis showed similar results. Fourth, there may be an issue with generalization. The frequency of HIV infection and condition of the pulmonary cavity were known risk factors affecting the clinical outcome and were considerably different among evaluated studies. Most studies were conducted in Africa, America, and India, and studies were rarely conducted in Asian countries and Russia despite a high prevalence of TB.

In conclusion, our systematic review of RCTs revealed that although the fluoroquinolone-containing regimen, including ofloxacin, gatifloxacin, levofloxacin, and moxifloxacin, resulted in a higher culture conversion rate at 2 months of treatment, it led to less favorable outcomes and more total adverse events compared with the standard regimen for drug-sensitive pulmonary TB. The results that suggest that the fluoroquinolone-containing regimen has limitations as the first-line therapy for pulmonary TB could help clinicians make treatment choices in everyday clinical practice.

## Supporting Information

S1 Fig Summary of the assessment of each risk of bias item (%) for each included study. (TIF)

S2 Fig Funnel plot of the sputum culture conversion at 2 months of treatment. (TIF)

S3 Fig Forest plot of the assessment of treatment failure. H = isoniazid; R = rifampicin; E = ethambutol; Z = pyrazinamide.
(TIF)

S4 Fig Forest plot of the assessment of hepatotoxicity. H = isoniazid; R = rifampicin; E = ethambutol; Z = pyrazinamide.
(TIF)

S5 Fig Forest plot of the assessment of drug rash and fever. H = isoniazid; R = rifampicin; E = ethambutol; Z = pyrazinamide.
(TIF)

S6 Fig Forest plot of the assessment of serious adverse events. H = isoniazid; R = rifampicin; E = ethambutol; Z = pyrazinamide.
(TIF)

S7 Fig Forest plot of the assessment of gastrointestinal adverse events. H = isoniazid; R = rifampicin; E = ethambutol; Z = pyrazinamide.
(TIF)

S8 Fig Forest plot of the assessment of dizziness. H = isoniazid; R = rifampicin; E = ethambutol; Z = pyrazinamide.
(TIF)

S9 Fig Forest plot of the assessment of joint pain. H = isoniazid; R = rifampicin; E = ethambutol; Z = pyrazinamide.
(TIF)

S10 Fig Forest plot of the assessment of peri-treatment mortality. H = isoniazid; R = rifampicin; E = ethambutol; Z = pyrazinamide.
(TIF)

S11 Fig Forest plot of the assessment of adherence to treatment. H = isoniazid; R = rifampicin; E = ethambutol; Z = pyrazinamide.
(TIF)

S1 File Details of the search strategy. (TIF)

S2 File The details of how to analyze the quality of methodology of the sources. (DOC)

S3 File Preferred reporting items for systematic review and meta-analysis checklist. (DOC)

## References

[1] Organization WH (2014) Global tuberculosis control: WHO report 2014. Geneva: World Health Organization.

[2] Combs DL, O'Brien RJ, Geiter LJ (1990) USPHS Tuberculosis Short-Course Chemotherapy Trial 21: effectiveness, toxicity, and acceptability. The report of final results. Ann Intern Med 112: 397–406. 2155569 10.7326/0003-4819-76-3-112-6-397

[3] Chaulk CP, Kazandjian VA (1998) Directly observed therapy for treatment completion of pulmonary tuberculosis: Consensus Statement of the Public Health Tuberculosis Guidelines Panel. Jama 279: 943–948. 9544769 10.1001/jama.279.12.943

[4] Cuneo WD, Snider DE Jr. (1989) Enhancing patient compliance with tuberculosis therapy. Clin Chest Med 10: 375–380. 2673646

[5] Karumbi J, Garner P (2015) Directly observed therapy for treating tuberculosis. Cochrane Database Syst Rev 5: Cd003343 10.1002/14651858.CD003343.pub4 26022367 PMC4460720

[6] East African and British Medical Research Councils (1978) Controlled clinical trial of five short-course (4-month) chemotherapy regimens in pulmonary tuberculosis. First report of 4th study. Lancet 2: 334–338. 79708

[7] Hong Kong Chest Service/British Medical Research Council (1991) Controlled trial of 2, 4, and 6 months of pyrazinamide in 6-month, three-times-weekly regimens for smear-positive pulmonary tuberculosis, including an assessment of a combined preparation of isoniazid, rifampin, and pyrazinamide. Results at 30 months. Am Rev Respir Dis 143: 700–706. 1901199 10.1164/ajrccm/143.4_Pt_1.700

[8] Pletz MW, De Roux A, Roth A, Neumann KH, Mauch H, Lode H (2004) Early bactericidal activity of moxifloxacin in treatment of pulmonary tuberculosis: a prospective, randomized study. Antimicrob Agents Chemother 48: 780–782. 14982764 10.1128/AAC.48.3.780-782.2004 PMC353154

[9] Hu Y, Coates AR, Mitchison DA (2003) Sterilizing activities of fluoroquinolones against rifampin-tolerant populations of Mycobacterium tuberculosis. Antimicrob Agents Chemother 47: 653–657. 12543673 10.1128/AAC.47.2.653-657.2003 PMC151758

[10] Chan ED, Laurel V, Strand MJ, Chan JF, Huynh ML, Goble M, et al (2004) Treatment and outcome analysis of 205 patients with multidrug-resistant tuberculosis. Am J Respir Crit Care Med 169: 1103–1109. 14742301 10.1164/rccm.200308-1159OC

[11] Goble M, Iseman MD, Madsen LA, Waite D, Ackerson L, Horsburgh CR Jr. (1993) Treatment of 171 patients with pulmonary tuberculosis resistant to isoniazid and rifampin. N Engl J Med 328: 527–532. 8426619 10.1056/NEJM199302253280802

[12] Kohno S, Koga H, Kaku M, Maesaki S, Hara K (1992) Prospective comparative study of ofloxacin or ethambutol for the treatment of pulmonary tuberculosis. Chest 102: 1815–1818. 1446494 10.1378/chest.102.6.1815

[13] Narayanan P (2002) Shortening short course chemotherapy: a randomised clinical trial for treatment of smear positive pulmonary tuberculosis with regimens using ofloxacin in the intensive phase. Indian J Tuberc 49: 27–38.

[14] Rustomjee R, Lienhardt C, Kanyok T, Davies GR, Levin J, Mthiyane T, et al (2008) A Phase II study of the sterilising activities of ofloxacin, gatifloxacin and moxifloxacin in pulmonary tuberculosis. Int J Tuberc Lung Dis 12: 128–138. 18230244

[15] Conde MB, Efron A, Loredo C, De Souza GRM, Graca NP, Cezar MC, et al (2009) Moxifloxacin versus ethambutol in the initial treatment of tuberculosis: a double-blind, randomised, controlled phase II trial. The Lancet 373: 1183–1189. 10.1016/S0140-6736(09)60333-0 PMC2866651 19345831

[16] Ziganshina LE, Titarenko AF, Davies GR (2013) Fluoroquinolones for treating tuberculosis (presumed drug-sensitive). Cochrane Database Syst Rev 6: Cd004795 10.1002/14651858.CD004795.pub4 23744519 PMC6532730

[17] Chen Z, Liang JQ, Wang JH, Feng SS, Zhang GY (2015) Moxifloxacin plus standard first-line therapy in the treatment of pulmonary tuberculosis: A meta-analysis. Tuberculosis (Edinb) 95: 490–496. 25964137 10.1016/j.tube.2015.03.014

[18] Jindani A, Harrison TS, Nunn AJ, Phillips PP, Churchyard GJ, Charalambous S, et al (2014) High-dose rifapentine with moxifloxacin for pulmonary tuberculosis. N Engl J Med 371: 1599–1608. 10.1056/NEJMoa1314210 25337749 PMC4233406

[19] Merle CS, Fielding K, Sow OB, Gninafon M, Lo MB, Mthiyane T, et al (2014) A four-month gatifloxacin-containing regimen for treating tuberculosis. N Engl J Med 371: 1588–1598. 10.1056/NEJMoa1315817 25337748

[20] Gillespie SH, Crook AM, McHugh TD, Mendel CM, Meredith SK, Murray SR, et al (2014) Four-month moxifloxacin-based regimens for drug-sensitive tuberculosis. N Engl J Med 371: 1577–1587. 10.1056/NEJMoa1407426 25196020 PMC4277680

[21] Jawahar MS, Banurekha VV, Paramasivan CN, Rahman F, Ramachandran R, Venkatesan P, et al (2013) Randomized clinical trial of thrice-weekly 4-month moxifloxacin or gatifloxacin containing regimens in the treatment of new sputum positive pulmonary tuberculosis patients. PLoS One 8: e67030 10.1371/journal.pone.0067030 23843980 PMC3700922

[22] Velayutham BV, Allaudeen IS, Sivaramakrishnan GN, Perumal V, Nair D, Chinnaiyan P, et al (2014) Sputum culture conversion with moxifloxacin-containing regimens in the treatment of patients with newly diagnosed sputum-positive pulmonary tuberculosis in South India. Clin Infect Dis 59: e142–149. 10.1093/cid/ciu550 25028463

[23] Liberati A, Altman DG, Tetzlaff J, Mulrow C, Gotzsche PC, Ioannidis JP, et al (2009) The PRISMA statement for reporting systematic reviews and meta-analyses of studies that evaluate health care interventions: explanation and elaboration. Ann Intern Med 151: W65–94. 19622512 10.7326/0003-4819-151-4-200908180-00136

[24] el-Sadr WM, Perlman DC, Matts JP, Nelson ET, Cohn DL, Salomon N, et al (1998) Evaluation of an intensive intermittent-induction regimen and duration of short-course treatment for human immunodeficiency virus-related pulmonary tuberculosis. Terry Beirn Community Programs for Clinical Research on AIDS (CPCRA) and the AIDS Clinical Trials Group (ACTG). Clin Infect Dis 26: 1148–1158. 9597244 10.1086/520275

[25] Burman WJ, Goldberg S, Johnson JL, Muzanye G, Engle M, Mosher AW, et al (2006) Moxifloxacin versus ethambutol in the first 2 months of treatment for pulmonary tuberculosis. Am J Respir Crit Care Med 174: 331–338. 16675781 10.1164/rccm.200603-360OC

[26] Dorman SE, Johnson JL, Goldberg S, Muzanye G, Padayatchi N, Bozeman L, et al (2009) Substitution of moxifloxacin for isoniazid during intensive phase treatment of pulmonary tuberculosis. Am J Respir Crit Care Med 180: 273–280. 10.1164/rccm.200901-0078OC 19406981

[27] Sirgel FA, Donald PR, Odhiambo J, Githui W, Umapathy KC, Paramasivan CN, et al (2000) A multicentre study of the early bactericidal activity of anti-tuberculosis drugs. J Antimicrob Chemother 45: 859–870. 10837441 10.1093/jac/45.6.859

[28] Wallis RS, Peppard T, Hermann D (2015) Month 2 culture status and treatment duration as predictors of recurrence in pulmonary tuberculosis: model validation and update. PLoS One 10: e0125403 10.1371/journal.pone.0125403 25923700 PMC4414505

[29] Cox H, Ford N, Keshavjee S, McDermid C, von Schoen-Angerer T, Mitnick C, et al (2011) Rational use of moxifloxacin for tuberculosis treatment. Lancet Infect Dis 11: 259–260. 10.1016/S1473-3099(11)70036-6 21453864

[30] Mitnick CD, McGee B, Peloquin CA (2009) Tuberculosis pharmacotherapy: strategies to optimize patient care. Expert Opin Pharmacother 10: 381–401. 10.1517/14656560802694564 19191677 PMC2674232

