# Supplementary figures and images for: The Effectiveness and Safety of Fluoroquinolone-Containing Regimen as a First-Line Treatment for Drug-Sensitive Pulmonary Tuberculosis: A Systematic Review and Meta-Analysis
Source: PLoS One. 2016 Jul 25;11(7):e0159827. doi: 10.1371/journal.pone.0159827 (PMC4959712; doi:10.1371/journal.pone.0159827)

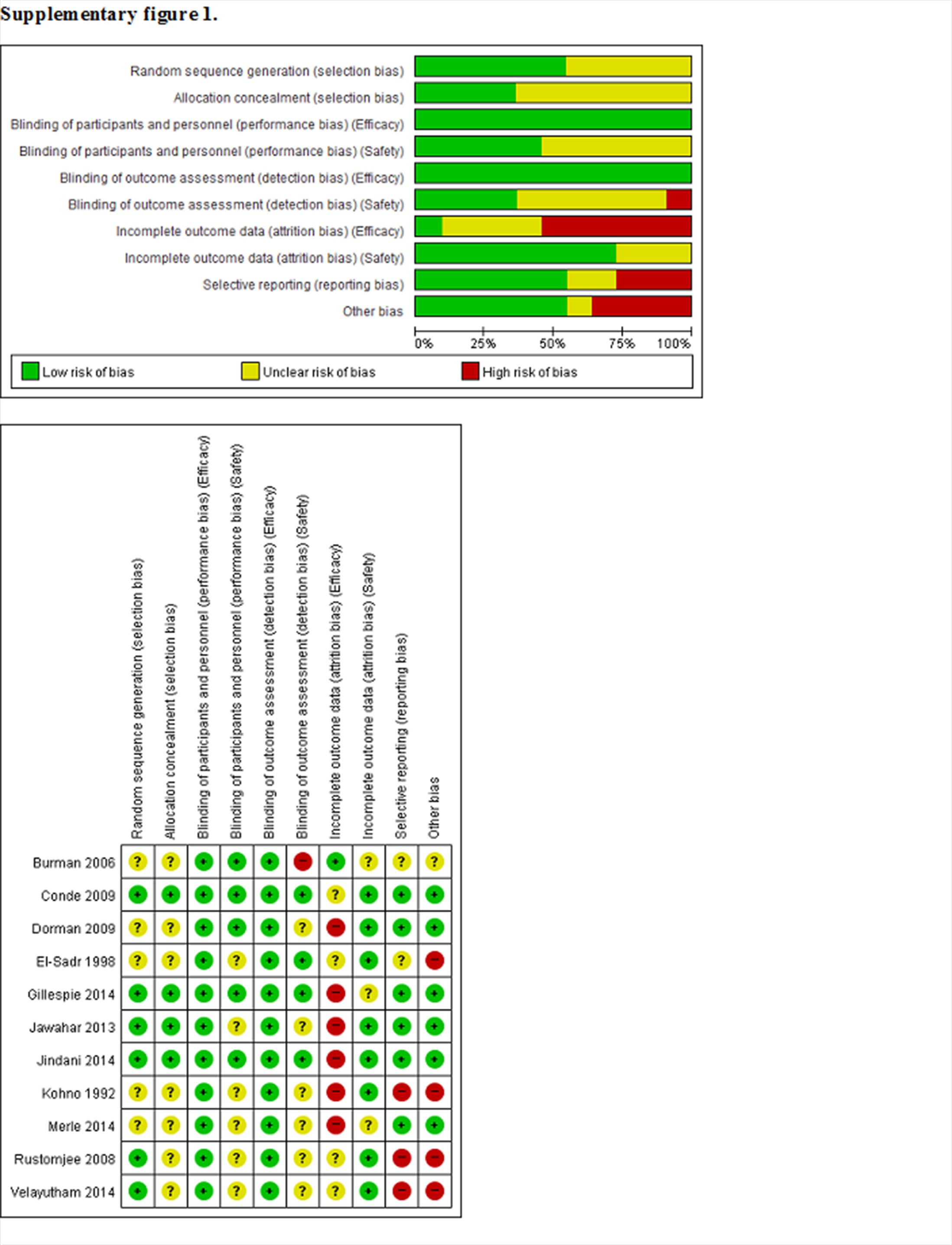

Supplement: S1 Fig — (TIF) [file pone.0159827.s001.tif]

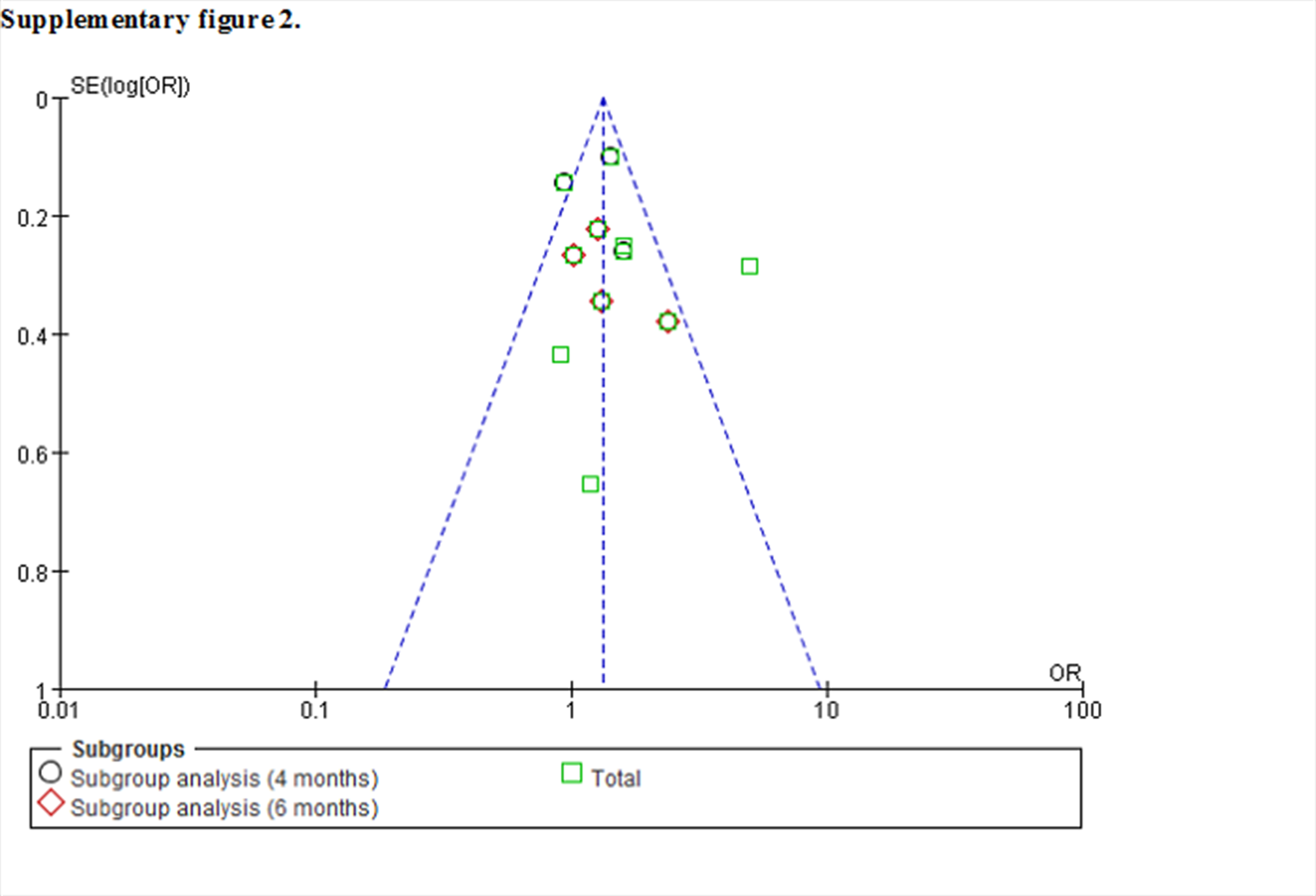

Supplement: S2 Fig — (TIF) [file pone.0159827.s002.tif]

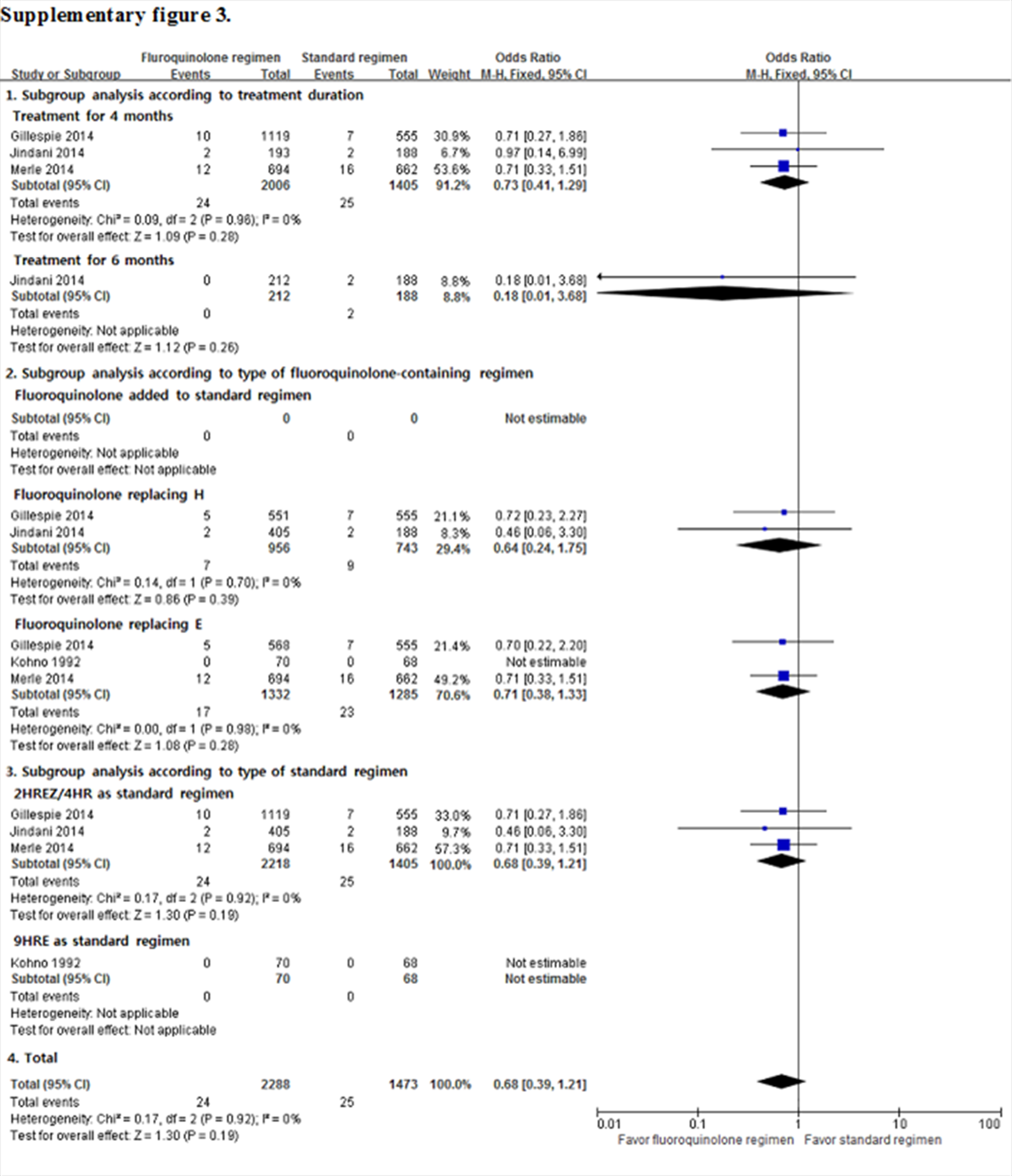

Supplement: S3 Fig — H = isoniazid; R = rifampicin; E = ethambutol; Z = pyrazinamide. (TIF) [file pone.0159827.s003.tif]

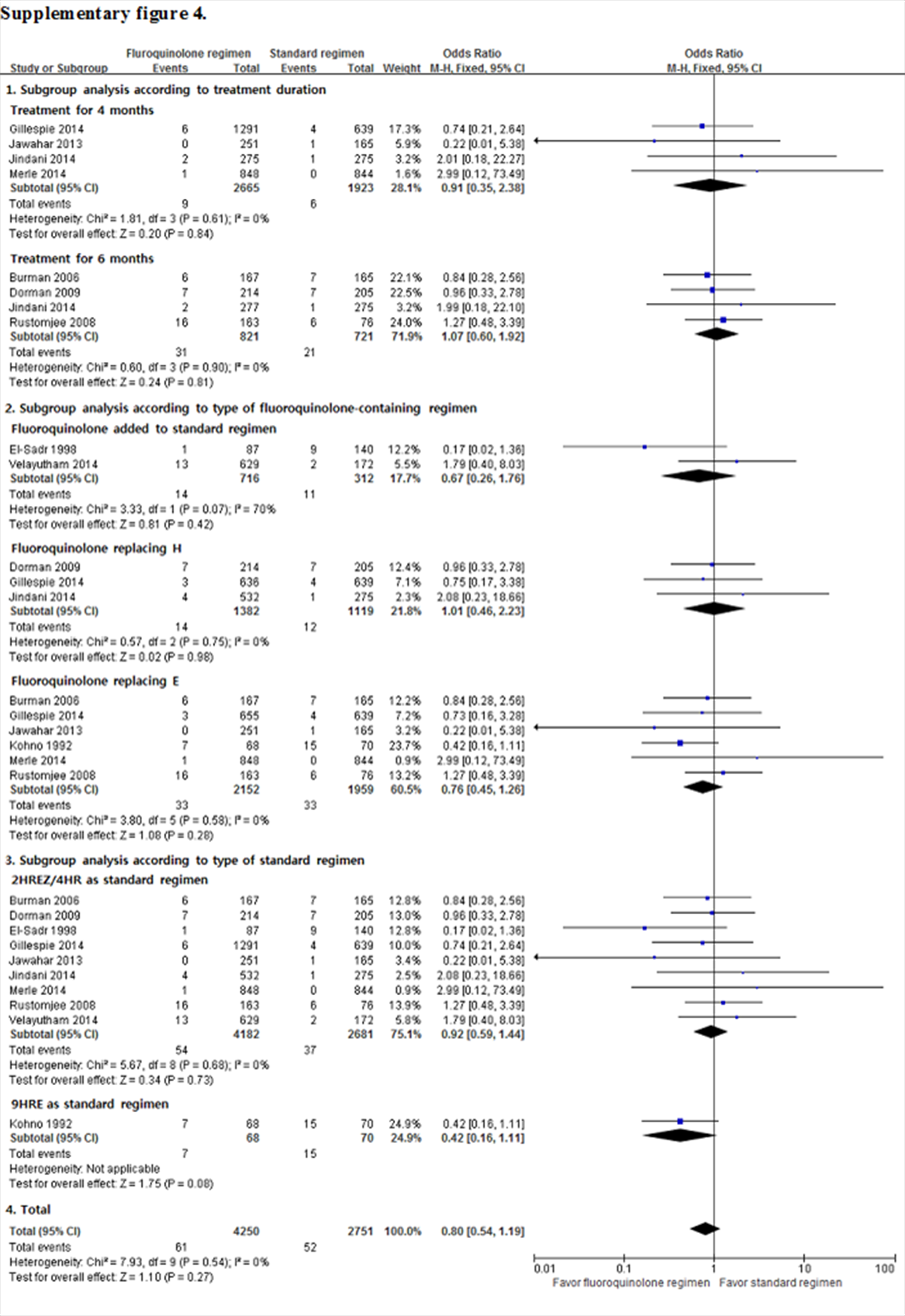

Supplement: S4 Fig — H = isoniazid; R = rifampicin; E = ethambutol; Z = pyrazinamide. (TIF) [file pone.0159827.s004.tif]

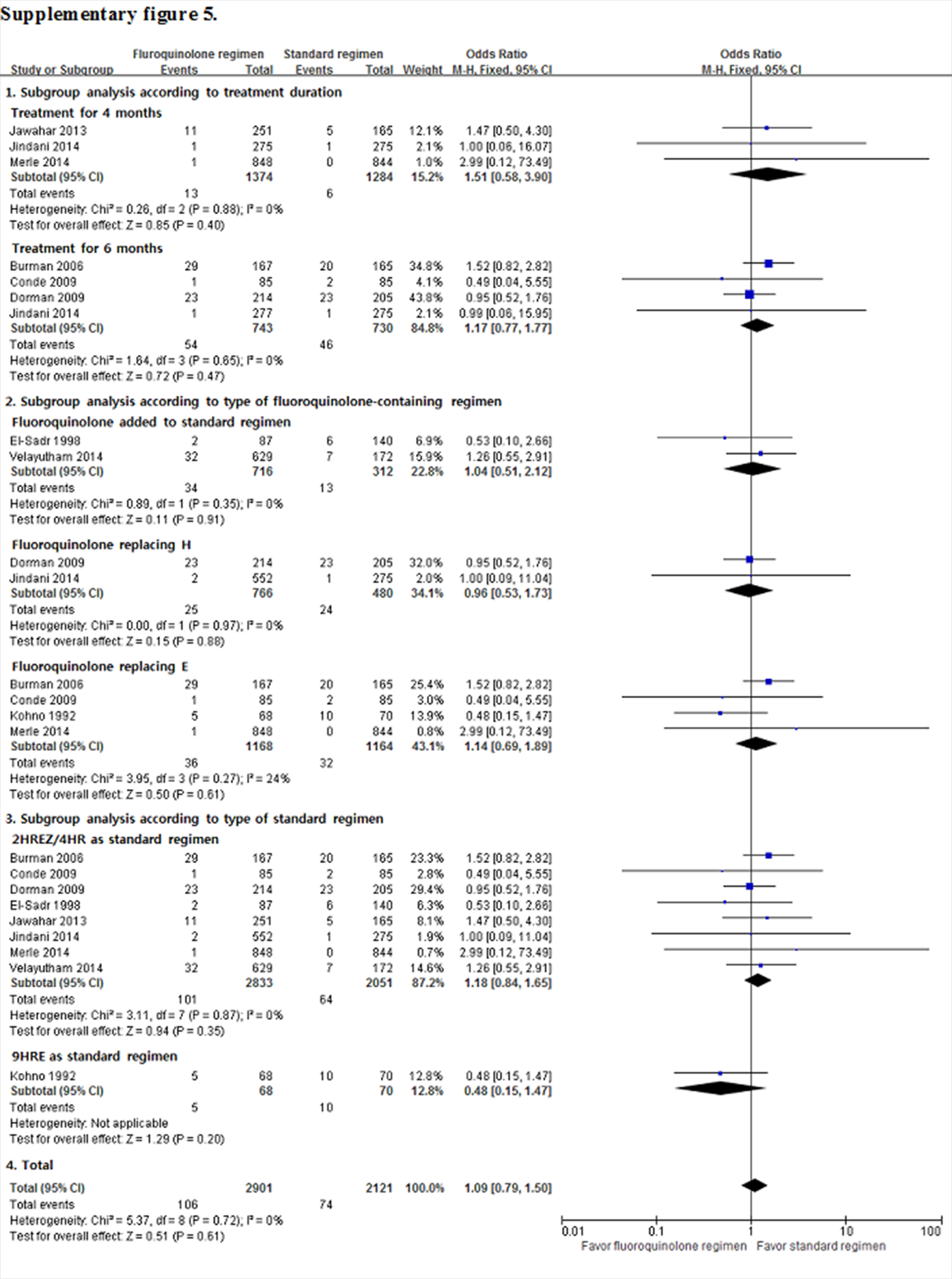

Supplement: S5 Fig — H = isoniazid; R = rifampicin; E = ethambutol; Z = pyrazinamide. (TIF) [file pone.0159827.s005.tif]

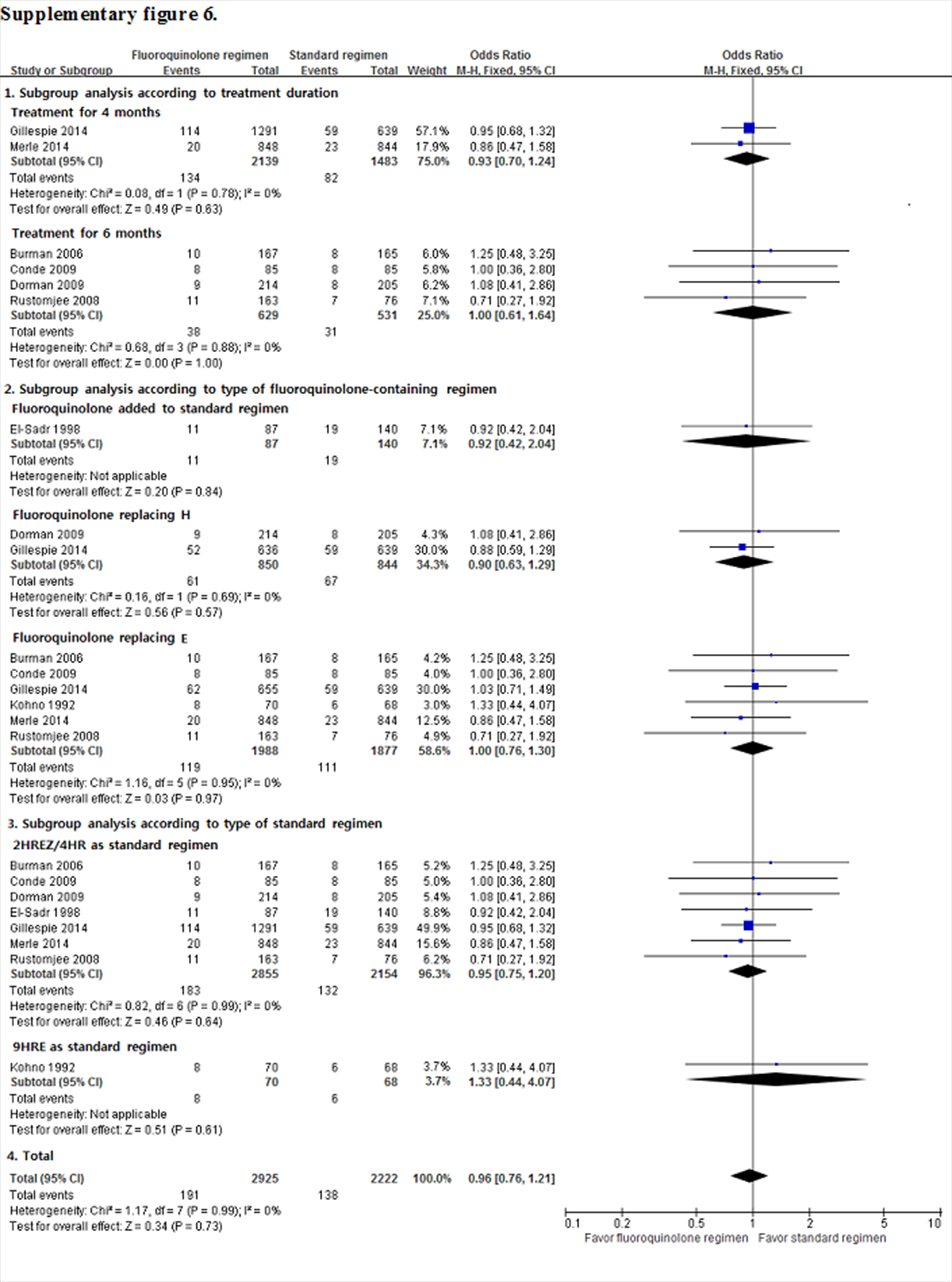

Supplement: S6 Fig — H = isoniazid; R = rifampicin; E = ethambutol; Z = pyrazinamide. (TIF) [file pone.0159827.s006.tif]

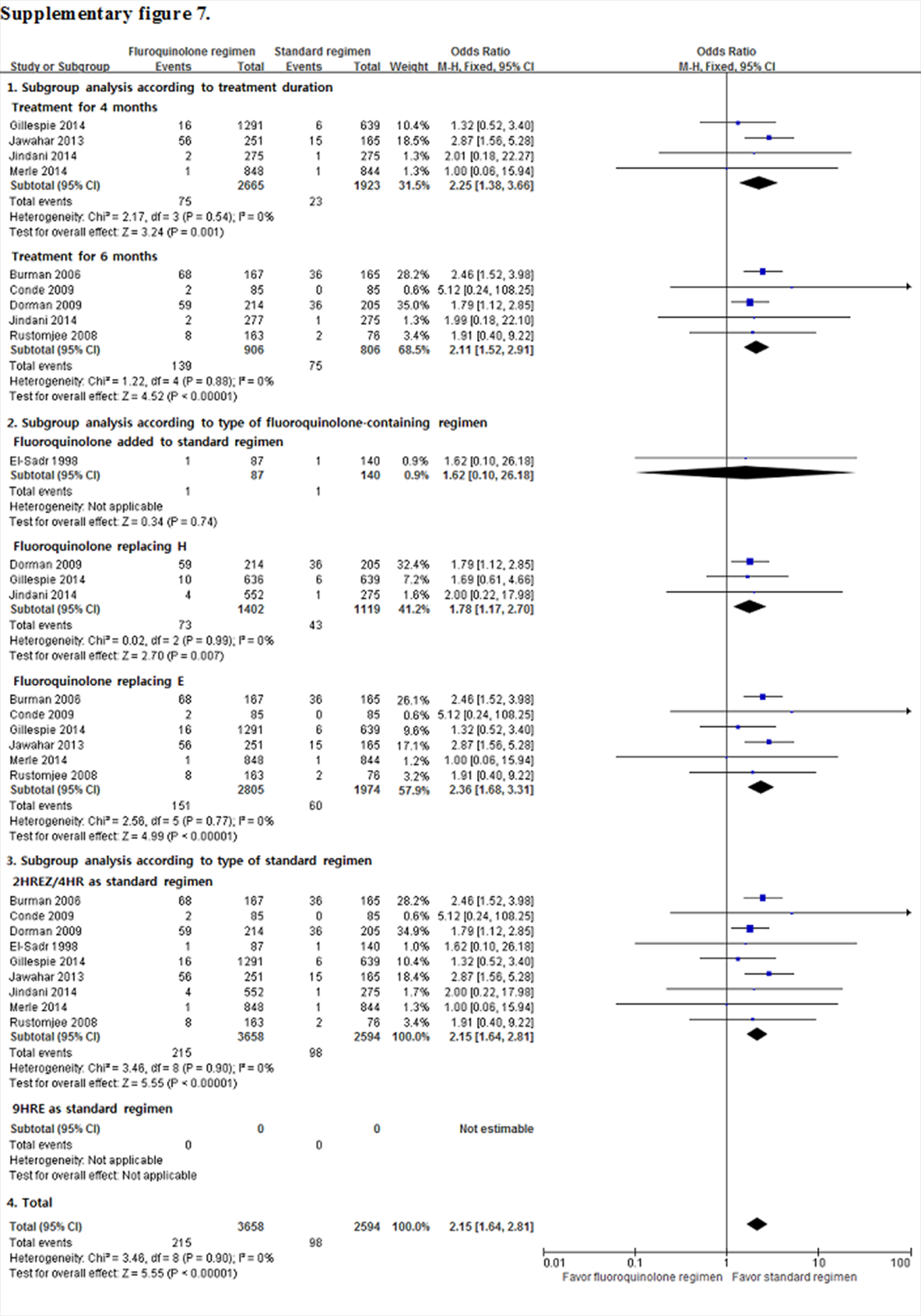

Supplement: S7 Fig — H = isoniazid; R = rifampicin; E = ethambutol; Z = pyrazinamide. (TIF) [file pone.0159827.s007.tif]

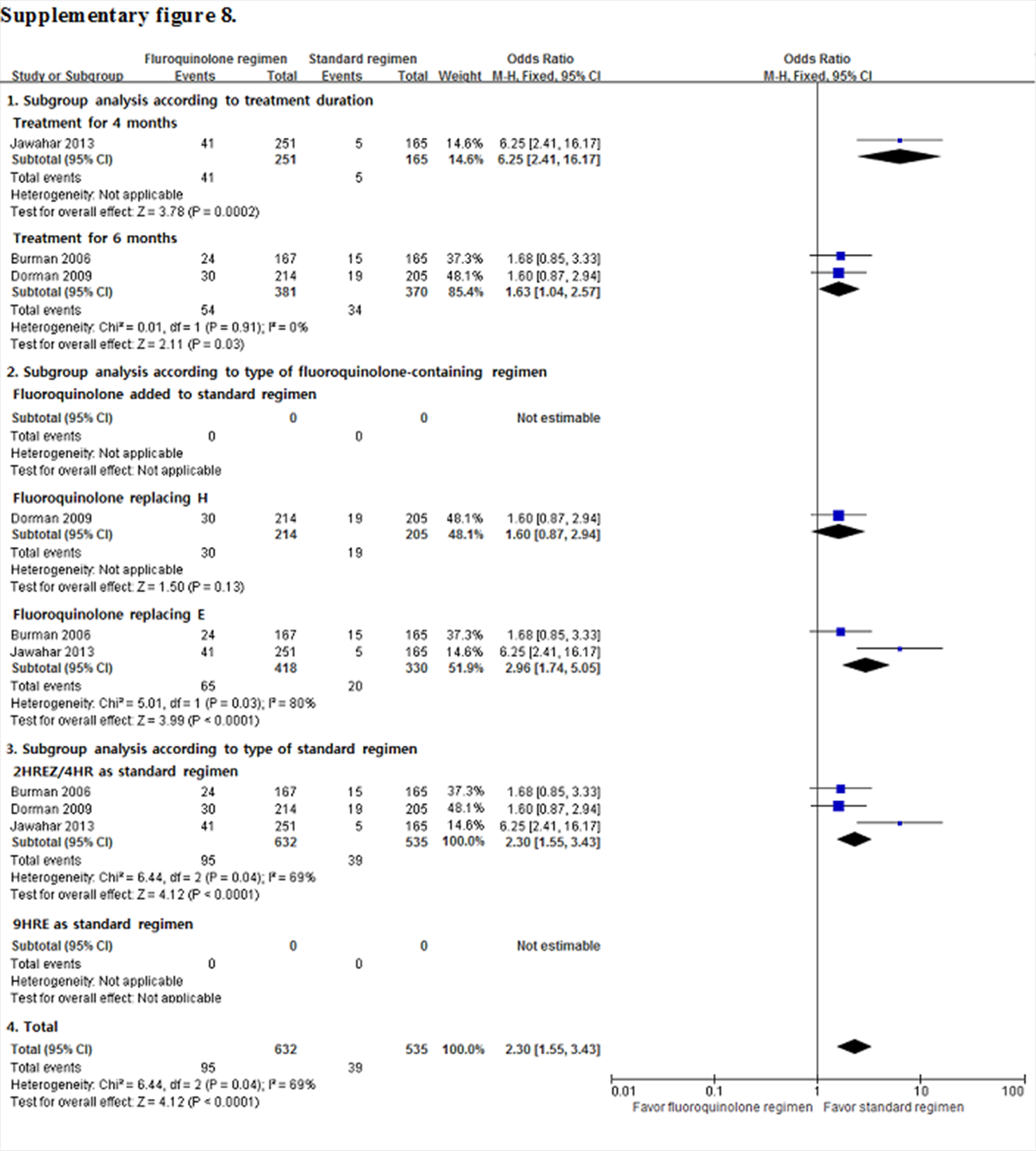

Supplement: S8 Fig — H = isoniazid; R = rifampicin; E = ethambutol; Z = pyrazinamide. (TIF) [file pone.0159827.s008.tif]

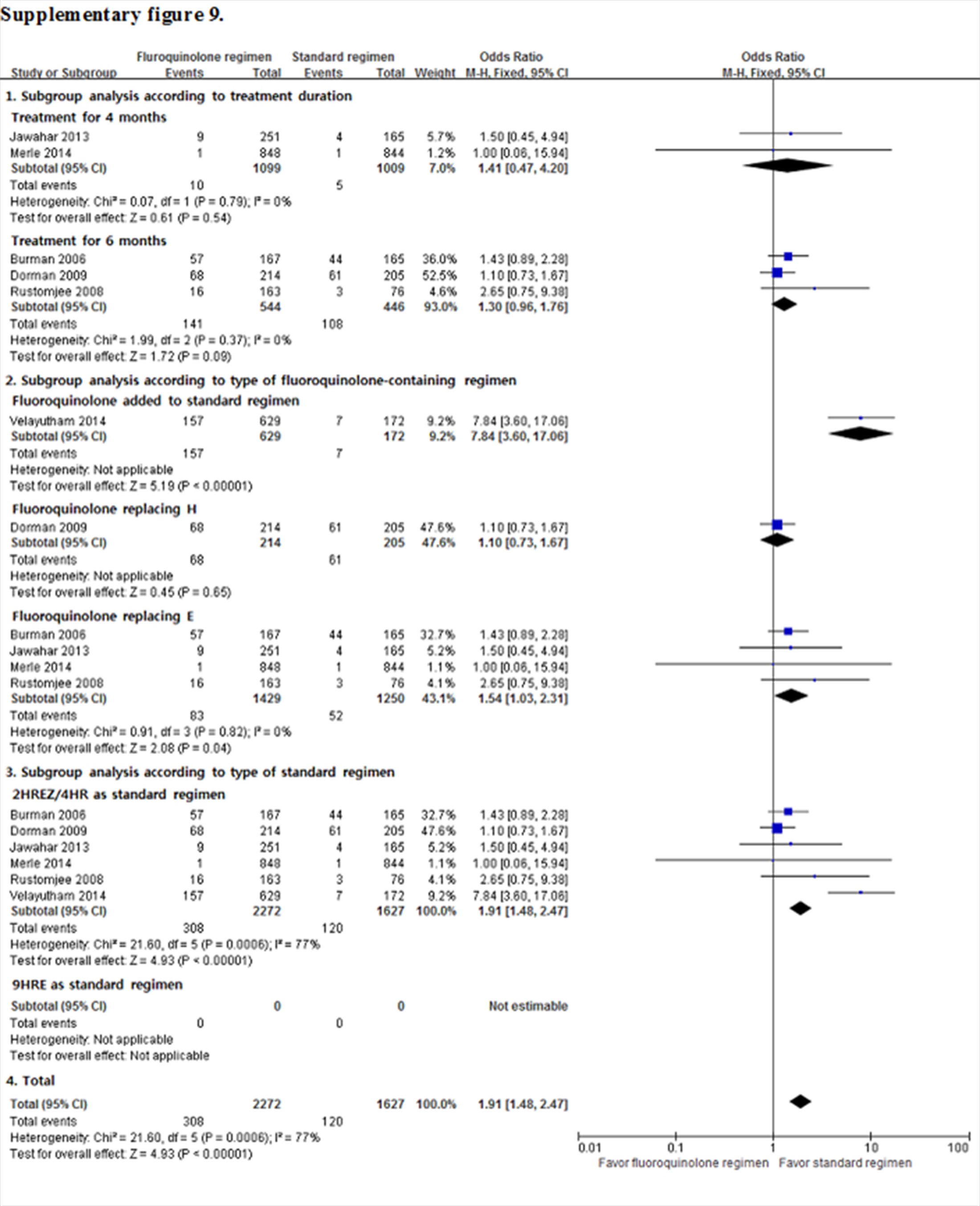

Supplement: S9 Fig — H = isoniazid; R = rifampicin; E = ethambutol; Z = pyrazinamide. (TIF) [file pone.0159827.s009.tif]

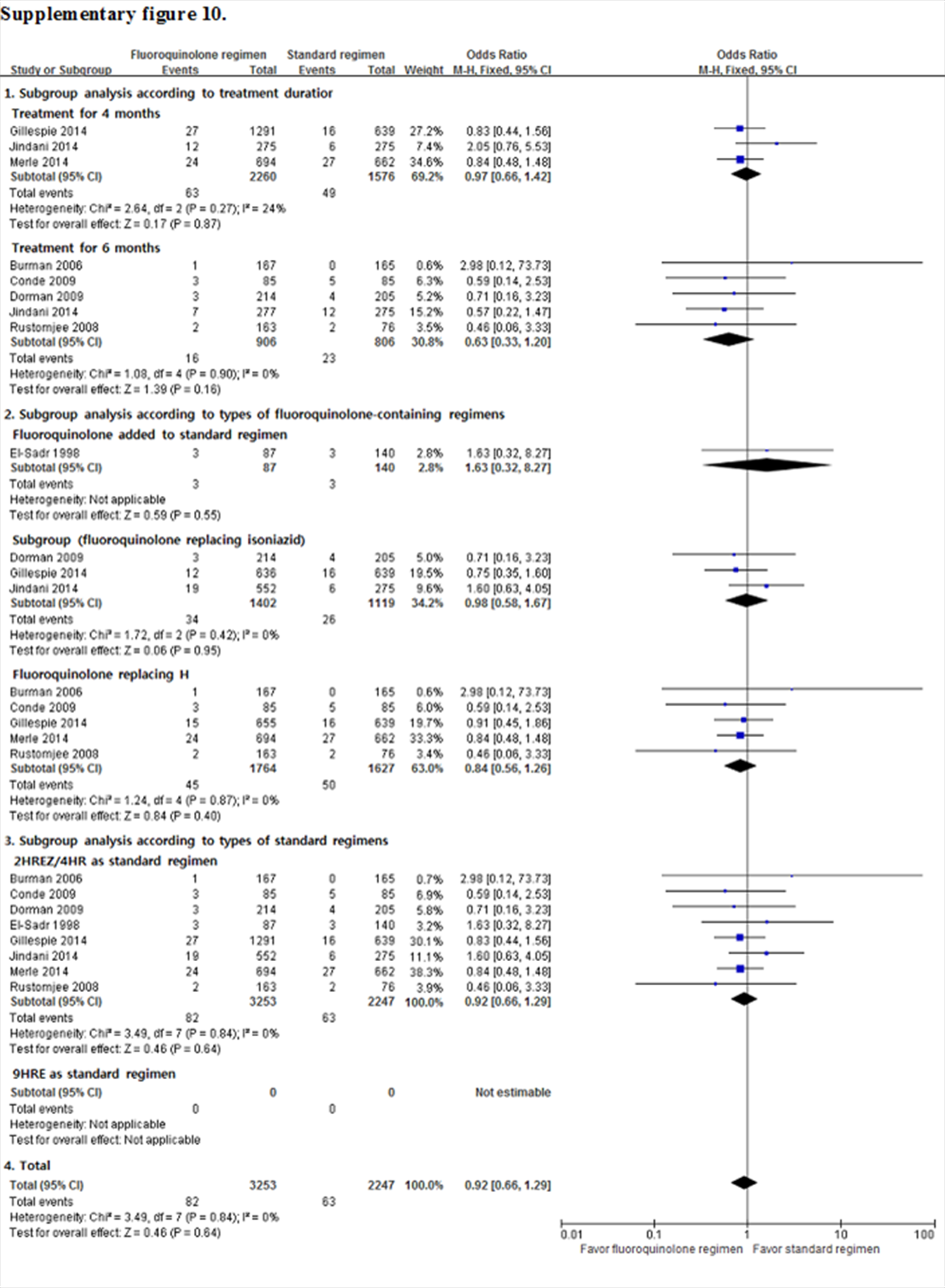

Supplement: S10 Fig — H = isoniazid; R = rifampicin; E = ethambutol; Z = pyrazinamide. (TIF) [file pone.0159827.s010.tif]

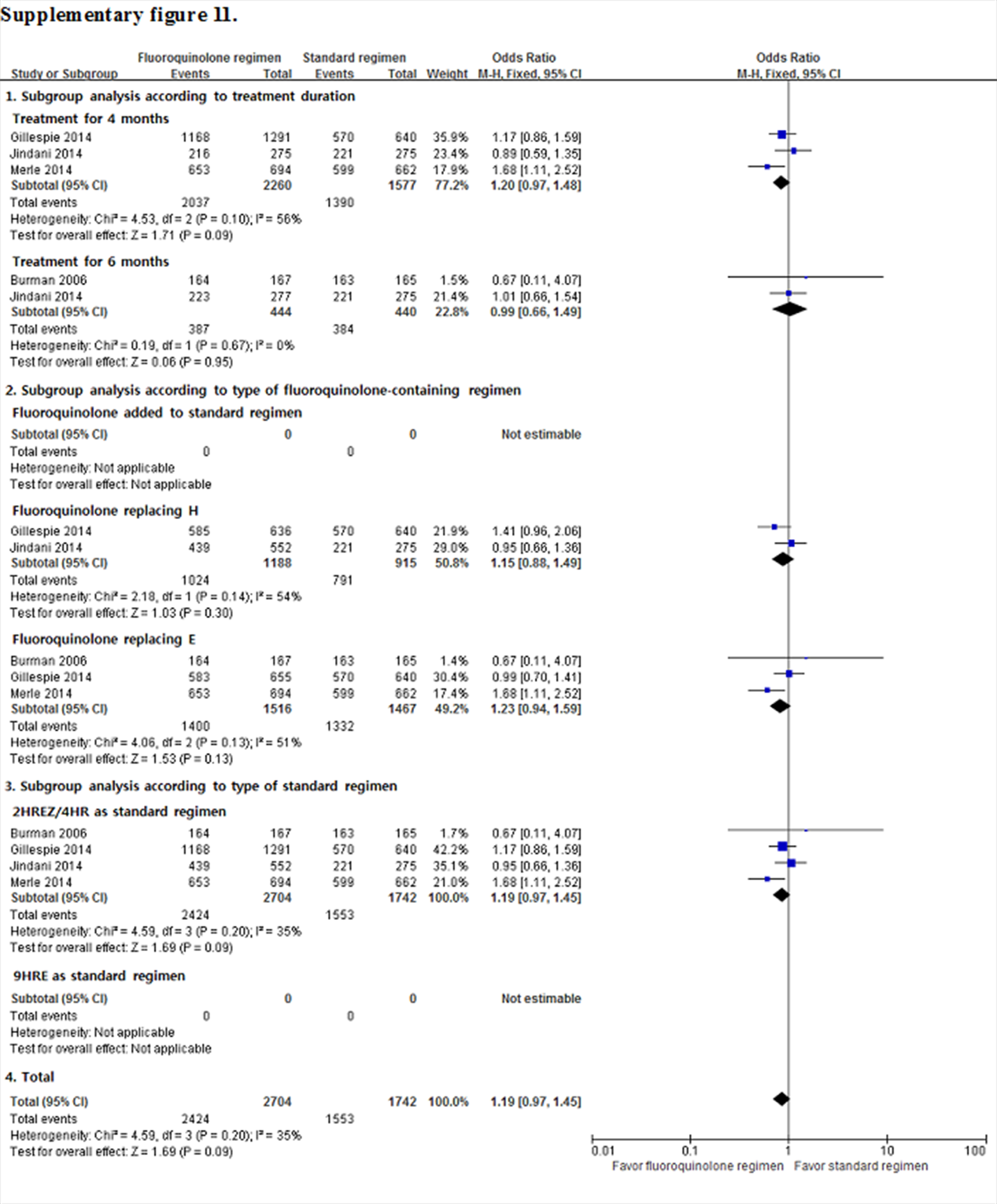

Supplement: S11 Fig — H = isoniazid; R = rifampicin; E = ethambutol; Z = pyrazinamide. (TIF) [file pone.0159827.s011.tif]

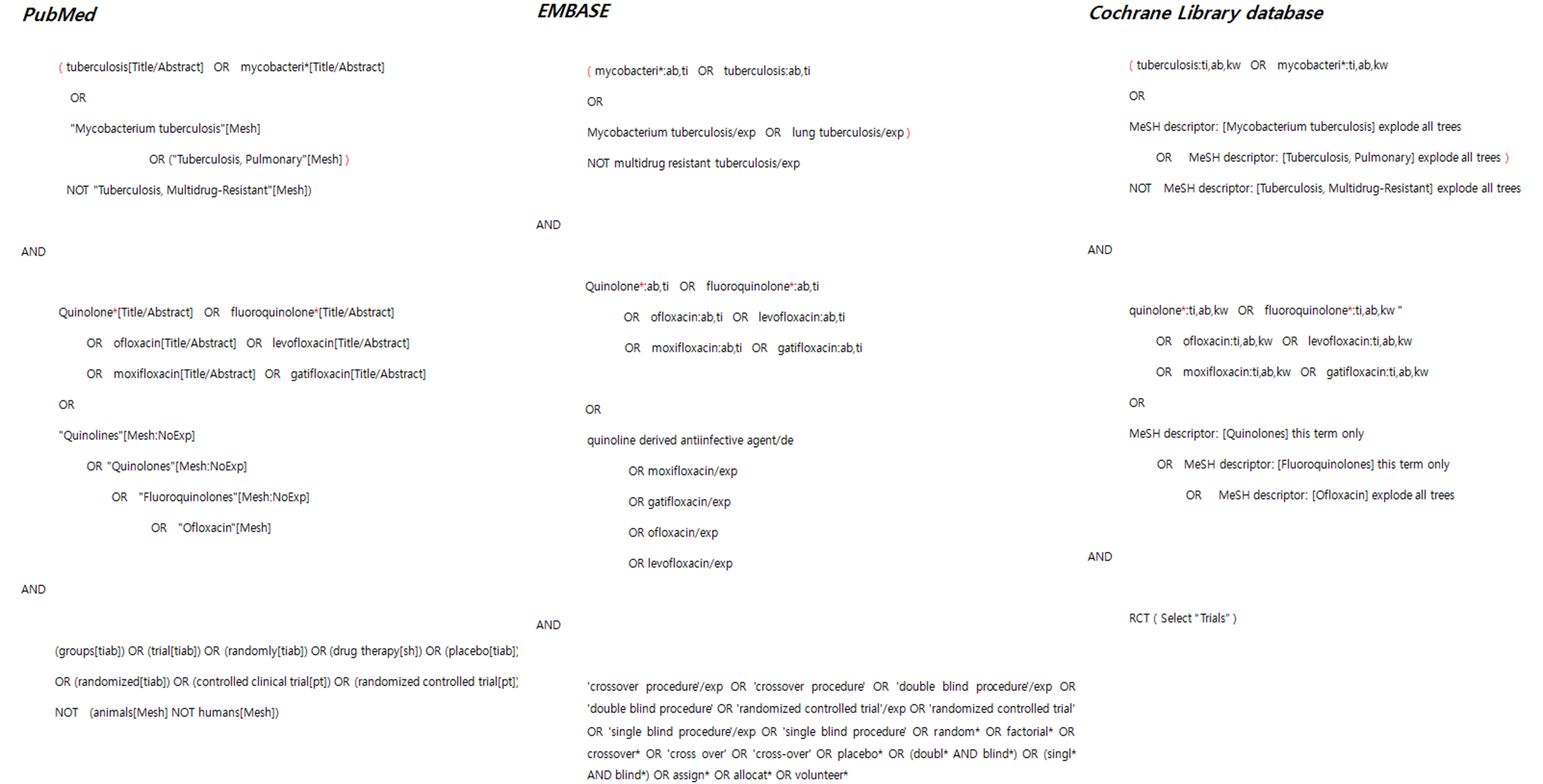

Supplement: S1 File — (TIF) [file pone.0159827.s012.tif]
