# Supplementary material for: The Effectiveness and Safety of Fluoroquinolone-Containing Regimen as a First-Line Treatment for Drug-Sensitive Pulmonary Tuberculosis: A Systematic Review and Meta-Analysis
Source: PLoS One. 2016 Jul 25;11(7):e0159827. doi: 10.1371/journal.pone.0159827 (PMC4959712; doi:10.1371/journal.pone.0159827)
Supplement: S2 File — (DOC) [file pone.0159827.s013.doc]

**Supplement file 2. The details of how to analyze the quality of methodology of the sources**

We assessed selection bias with random sequence generation and allocation concealment, which were mentioned in the articles. Classification of the random sequence generation or allocation concealment into unclear risk of bias was not mentioned. Performance bias was evaluated with effective blinding of the participants and researchers. We assessed performance bias in the two aspects of efficacy and safety. In the evaluation of efficacy outcome, blinding was not necessary to ensure a low risk of bias. An invalid method of blinding about safety was considered a high risk of bias. The absence of a remark about blinding was considered an unclear risk of bias. We evaluated attrition bias by using incomplete outcome data in the two aspects of efficacy and safety. Low risk of bias was assumed in the following cases: no missing data, the reason of exclusion after randomization was not relevant to the study result, the number and reason of the missing data were similar between the regimens, and appropriate analysis was performed. Reporting bias was assessed by reviewing the study protocol and searching any missing result that could influence the conclusion of the study. If a study protocol was not available, we assessed the study as having an unclear risk of bias.

A funnel plot analysis was also performed to exclude publication bias. Other biases were primarily assessed based on any problems in study design, differences in baseline characteristics, or financial conflict of interest. If patients with TB resistant to rifampin or fluoroquinolone were found in the course of a study, we assessed the study as having a high risk of bias.
